# Higher-Order Conditioning and Dopamine: Charting a Path Forward

**DOI:** 10.3389/fnbeh.2021.745388

**Published:** 2021-10-04

**Authors:** Benjamin M. Seitz, Aaron P. Blaisdell, Melissa J. Sharpe

**Affiliations:** Department of Psychology, University of California, Los Angeles, Los Angeles, CA, United States

**Keywords:** dopamine, sensory preconditioning, second order conditioning, reinforcement learning, basolateral amygdala, hippocampus, orbitofrontal cortex

## Abstract

Higher-order conditioning involves learning causal links between multiple events, which then allows one to make novel inferences. For example, observing a correlation between two events (e.g., a neighbor wearing a particular sports jersey), later helps one make new predictions based on this knowledge (e.g., the neighbor’s wife’s favorite sports team). This type of learning is important because it allows one to benefit maximally from previous experiences and perform adaptively in complex environments where many things are ambiguous or uncertain. Two procedures in the lab are often used to probe this kind of learning, second-order conditioning (SOC) and sensory preconditioning (SPC). In second-order conditioning (SOC), we first teach subjects that there is a relationship between a stimulus and an outcome (e.g., a tone that predicts food). Then, an additional stimulus is taught to precede the predictive stimulus (e.g., a light leads to the food-predictive tone). In sensory preconditioning (SPC), this order of training is reversed. Specifically, the two neutral stimuli (i.e., light and tone) are first paired together and then the tone is paired separately with food. Interestingly, in both SPC and SOC, humans, rodents, and even insects, and other invertebrates will later predict that both the light and tone are likely to lead to food, even though they only experienced the tone directly paired with food. While these processes are procedurally similar, a wealth of research suggests they are associatively and neurobiologically distinct. However, midbrain dopamine, a neurotransmitter long thought to facilitate basic Pavlovian conditioning in a relatively simplistic manner, appears critical for both SOC and SPC. These findings suggest dopamine may contribute to learning in ways that transcend differences in associative and neurological structure. We discuss how research demonstrating that dopamine is critical to both SOC and SPC places it at the center of more complex forms of cognition (e.g., spatial navigation and causal reasoning). Further, we suggest that these more sophisticated learning procedures, coupled with recent advances in recording and manipulating dopamine neurons, represent a new path forward in understanding dopamine’s contribution to learning and cognition.

## Dopamine and Higher-Order Cognition: Charting a Path Forward

### Introduction

To understand their worlds, humans and other animals learn to predict outcomes that are important to them, like food or pain. This is adaptive; if you can predict these outcomes, you can learn to increase or decrease your chances of encountering them depending on current needs. Sometimes this process is simple. The sight of a burrito predicts calories. But often it is more complex. Perhaps you have to remember the name of the restaurant that sells the burrito, or even recall the route you previously took to get there. This more complex learning process is referred to as higher-order conditioning and involves the combining of information that allows one to navigate cognitively or spatially to their goals. Higher-order conditioning likely accounts for many of our learned experiences; learning how to predict the consequences of our environment is rarely a more simplistic encounter with direct predictors of food or pain (Gewirtz and Davis, 2000).

**Figure 1:**
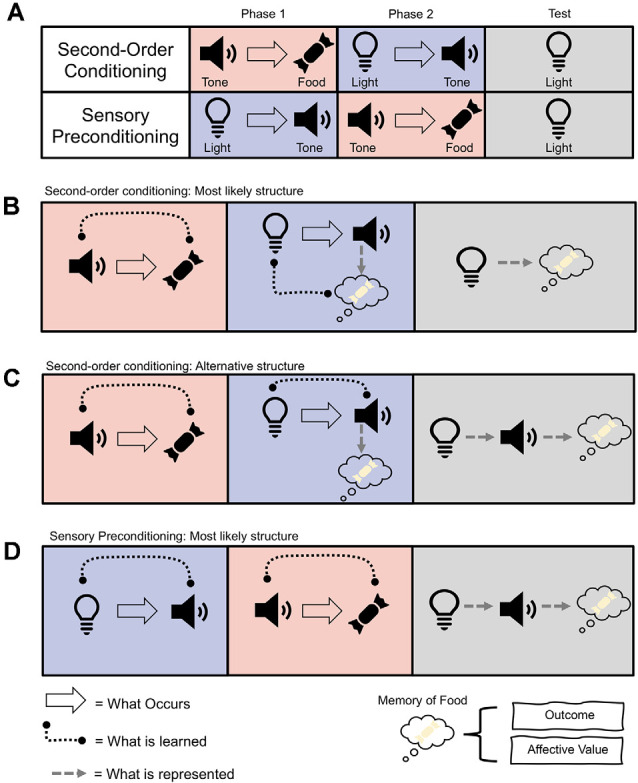
**(A)** Example procedures for second-order conditioning (SOC) and sensory preconditioning (SPC). **(B)** Most likely associative structure underlying SOC, whereby the light becomes directly associated with a memory of the food and its affective value or sensory-specific representation. **(C)** Alternative associative structure of SOC whereby light is associated with the tone, which is associated with the memory of the food and induces a response. **(D)** Most likely associative structure of SPC whereby the light is associated with the tone, which is associated with a memory of the food and involves the hippocampus and orbitofrontal cortex (OFC).

In the lab, we mimic this process of higher-order conditioning through the use of the second-order conditioning (SOC) and sensory preconditioning (SPC) procedures. SOC was first described by Pavlov (1927) and refers to instances in which a neutral stimulus (e.g., a tone) is paired with something important, like food. After this, another novel stimulus (e.g., a light) is paired with the tone. SOC occurs when the light elicits an appetitive response by virtue of being paired with the food-predictive tone (see Figure 1A). Thus, because the tone has been directly paired with reward, it can now reinforce associations between itself and stimuli that predict it (i.e., the light). On the other hand, SPC involves first pairing the light and tone together when they are both neutral and then presenting the tone with something significant (e.g., food). SPC refers to the finding that humans and other animals will now show an appetitive response to the light, even though they have never experienced the light directly paired with food (Brogden, 1939; see Figure 1A). These procedures indicate that we can learn complex mental routes to something biologically significant, even if what we are learning about has not been directly paired with those significant outcomes.

At first glance, the phenomena of SOC and SPC might seem similar. Indeed, the only difference in their procedures is the order of training (Figure 1A). That is, both procedures involve pairing two neutral stimuli together, the light and the tone, and separately pairing the tone with food. Yet in SOC the pairing of the neutral stimuli occurs after pairings of the tone with food, and in SPC the pairing of the neutral stimuli occurs before pairings of the tone with food. Despite this seemingly minor difference, SOC and SPC differ in their associative structure and neural substrates. SOC appears to rely on the transfer of affective value from the food-predictive tone to the light, facilitated by amygdala circuits (Gewirtz and Davis, 1997; Parkes and Westbrook, 2010). In contrast, SPC relies on forming a more complex association between all three elements (i.e., light→tone→food), with help from the hippocampus and orbitofrontal cortex (Rizley and Rescorla, 1972; Jones et al., 2012; Barron et al., 2020; Hart et al., 2020). Phasic dopamine activity in the midbrain, however, has recently been shown to be necessary for both phenomena to occur (Sharpe et al., 2017a; Maes et al., 2020). This places midbrain dopamine (and the dopamine prediction error, described below) at the heart of more complex learning and cognition. Herein, we review how dopamine unites the contrasting processes of SOC and SPC, and the implications this has for conceptualizing dopamine as a teaching signal that transcends associative structure.

### Associative Structure and Neural Substrates of Higher-Order Conditioning

The finding that a stimulus (e.g., tone) paired with reward (e.g., food) can on its own come to elicit a response (e.g., a rat making a nose poke into the location where food is delivered) seems a straightforward and obvious phenomenon. But over a century of research in Pavlovian conditioning has revealed that diagnosing the associative basis of behavior is not necessarily straightforward. For instance, the tone might be associated with the specific response to nose poke. Here, the presentation of the tone automatically causes the animal to nose poke in a reflexive manner (i.e., a tone-response association). On the opposite side of the spectrum, the tone might be associated with a detailed and rich sensory-specific memory of the food. In this scenario, the presentation of the tone makes the animal think about the food outcome and its various features (e.g., texture, odor, and taste), and thinking about this specific outcome drives the response to nose poke towards the location where food is usually delivered (i.e., a tone-outcome association). Somewhere in between these two accounts, the tone may become associated with the general affective value of the food reward, and this appetitive value may drive the nose poking response (i.e., a tone-value association). One tool learning theorists have used to differentiate between these accounts involves manipulating the representation or desirability for the outcome (Rescorla and Solomon, 1967; Dickinson, 1985; Dolan and Dayan, 2013). To use the example above, if the food was paired with illness (referred to as “devaluation”), an animal that has associated the tone with a detailed representation of the outcome will recall that they no longer find the food rewarding and will not nose poke at the location. However, if the animal has learned a more reflexive association between the tone and nose poking, or the tone and the appetitive value, devaluation of the outcome should not influence nose poking. This is because the association involves the tone and the response or value, not tone and the specific food outcome it predicts. Effectively, the ability of a stimulus to drive a behavioral response may originate from many different associations. Indeed, these associations may even drive the response at the same time (Rescorla, 1988). Accordingly, we need to adequately test and probe the associative basis of any given association to understand its underlying structure and neural substrates.

Below, we review the associative and neural basis of SOC and SPC. For simplicity, we will continue to use our example where the tone is directly paired with the outcome (food or shock), and the light predicts the tone. However, this does not necessarily reflect the stimuli used in the procedures discussed. Indeed, SOC and SPC are not limited to conditioning with food or pain, nor do they require such simplistic stimuli to occur. SOC and SPC have been observed repeatedly using a number of different procedures, complex stimuli (e.g., spatial landmarks), and species ranging from sea slugs (Hawkins et al., 1998) to pigeons (Sawa et al., 2005) to humans (Wimmer and Shohamy, 2012; Craddock et al., 2018). We also note that some of the to-be-reviewed structures have only had their involvement tested in one phenomenon (e.g., orbitofrontal cortex in SPC) and may or may not be involved in the other.

#### Second-Order Conditioning

SOC allows for predictive stimuli to facilitate further learning to neutral stimuli that precede it. For example, once the tone has been established as predictive of food, it can reinforce the development of an appetitive association with the light that now predicts its occurrence. There are several associative structures that could support this learning (see Figures 1B,C). The first possibility is an association between the light and tone, whereby the presentation of the light elicits a representation of tone, which then elicits a memory of the food resulting in the conditioned response (Rizley and Rescorla, 1972; Barnet et al., 1997). This is said to be a more cognitive account because it relies on the light evoking a representation of the tone. The second possibility is a more direct association between light and the food (Konorski, 1967). According to this view, the tone evokes a representation of the food, and so when the light is paired with the tone, it too becomes associated with the representation of food.

To test these two accounts, researchers have manipulated the status (i.e., memory) of the tone after it is paired with the light (i.e., tone→food, light→tone), but before the light is tested alone to assess the magnitude of SOC. For example, Rizley and Rescorla (1972) repeatedly presented the tone without consequence after establishing the tone-light association. This process of extinction reduced responding to tone. However, the light still elicited the same magnitude of SOC (for a recent replication in rats, see Holmes et al., 2014; and in humans see Jara et al., 2006; but for failed replications in humans and discussion, see Craddock et al., 2018; Lee, 2021). Similarly, Holland and Rescorla (1975) devalued the food outcome after establishing the tone→food and light→tone associations. Here, devaluation also attenuated responding the tone, while responding to the light remained intact. These results suggest responding to light does not rely on an evoked representation of tone, or a sensory-specific representation of food.

Further insight into the associative structure of SOC is provided by the fact that light and tone can exhibit different types of responses. For example, when pigeons learn that a tone predicts food, presentation of the tone elicits general food-seeking behavior towards the location of where the food is delivered. However, when light is paired with food, pigeons will peck at the source of the light (i.e., a key; Nairne and Rescorla, 1981). In SOC, when the light is paired with the food-predictive tone, the light will still evoke the key peck. Thus, SOC does not seem to be supported by an association between the light and the conditioned response evoked by the tone (Gewirtz and Davis, 2000). A more conservative summary of the data, therefore, is that responding to the light in SOC is associated with an affective state—or valence—but it does not evoke a representation of the tone or the response associated with the tone (see Figure 1B; Holland, 1977; Gewirtz and Davis, 2000).

The neural regions that are involved in SOC make understanding of the associative nature more complex. In particular, studies (e.g., Holmes et al., 2013) have shown that glutamatergic signaling in the basolateral amygdala (BLA), likely facilitated by BLA pyramidal neurons, is necessary for SOC in an aversive setting. That is, infusion of an NMDA antagonist (AP5 or ifenprodil) prior to the pairing of the light with a shock-predictive tone, prevents the ability of the light to support SOC. This is contradictory to the hypothesis that SOC relies on the transfer of general valence to the light as the BLA is known to be critical for the development of sensory-specific associations between stimuli and outcomes, and explicitly not associations between stimuli and general value (Corbit and Balleine, 2005; Balleine and Killcross, 2006; Prévost et al., 2012). Thus, it is surprising that BLA is necessary for the development of SOC in this phenomenon. This may suggest either SOC is not the result of the transfer of general valence, or indicate the presence of multiple associations driving SOC, with some aspects and/or procedures being supported by the BLA.

In support of this, the BLA appears less critical in SOC with appetitive reinforcement. For example, lesions to the BLA before the tone is paired with food will prevent the development of SOC when the light is subsequently paired with the food-predictive tone. However, if similar lesions are made after the tone is established as food-predictive and before the light is paired with the tone, SOC is spared (Setlow et al., 2002) and in some instances, enhanced (Holland, 2016). The reason for the discrepancy in BLA involvement between aversive and appetitive SOC might rest in the amount of training that supports learning in aversive and appetitive procedures. Aversive procedures generally use few pairings of stimuli and aversive outcomes, while appetitive procedures involve many pairings of stimuli and outcomes, across days or even weeks. Holland has shown that if the tone is paired with food across few pairings, the tone will be able to serve as a “substitute” for the food. For example, if the tone is devalued (i.e., paired with LiCl) the food will now also be devalued (i.e., mediated conditioning). However, if the tone is paired many times with food, it will no longer substitute as the food in mediated conditioning, despite the tone still producing an appetitive response (Holland, 1998). This could suggest that the number of pairings of the tone and outcome might influence the nature of the association that is supported during SOC. Accordingly, the general value may be sufficient to support SOC in appetitive procedures, which generally utilize many pairings of the tone and outcome, making the BLA unnecessary. In contrast, the associations driving SOC in aversive conditioning may be more based on associations between stimuli and detailed representations of outcomes and require the BLA, which encodes these forms of associations (Balleine and Killcross, 2006; Wassum and Izquierdo, 2015). Of course, this hypothesis is yet to be tested and it is possible that other differences between appetitive and aversive SOC procedures could underlie this discrepancy. However, it is unlikely to be the general appetitive or aversive nature of the task *per se*, as many researchers have found BLA plays a similar role in learning about food and shocks (Balleine and Killcross, 2006; Wassum and Izquierdo, 2015).

Similarly, to the role of the BLA in SOC, the role of the hippocampus in SOC is mixed. Lin and Honey (2011) found SOC was unaffected by pre-training lesions encompassing the dorsal and ventral hippocampus. On the other hand, Gilboa et al. (2014) found that these pre-training lesions prevented SOC, while the response to the food-predictive tone remained intact. However, their SOC procedure was a bit unorthodox in that after pairing the tone with food, they then paired the tone with the light (typically light is paired with tone). According to most accounts of value transfer [e.g., Temporal Difference Reinforcement Learning, see Sutton and Barto (1981)], this procedure is likely to occlude the transfer of value from the tone to the light because the value in these models is thought to back propagate to earlier predictors of reward. Thus, presenting the food predictive tone followed by light may have “forced” a more cognitive associative structure of SOC and thus relied on the hippocampus.

Interestingly, the retrosplenial cortex, a brain region that projects to (and receives information from) the hippocampus and that is known to be involved in learning and memory processes (Bucci and Robinson, 2014), also does not appear necessary for SOC (Todd et al., 2016). Ultimately, the fact that SOC may in some instances be reliant on the hippocampus but in other instances be hippocampal-independent, may again reflect the fact that SOC can be supported by several different types of associations (see Figures 1B,C). Findings of hippocampal involvement in SOC might depend on certain SOC procedures that encourage associations between the light and tone, or light and food, whereas those that suggest the hippocampus and retrosplenial cortex are not involved in SOC might derive from procedures that favor the light and valence of the outcome.

#### Sensory Preconditioning

SPC involves first presenting the light and tone together and then pairing the tone with food (or another outcome), which results in an appetitive response being elicited by both the light and tone. In this way, SPC can be taken as the strongest evidence in favor of animals learning associations between truly neutral stimuli, as neither stimulus was motivationally significant prior to their pairing. Unlike the mixed data that investigates the associative basis of SOC, it is reasonably well accepted that SPC entailed a cognitive representation between the light, tone, and outcome, which have been chained together by the inference that the light is likely to lead to food as its associate, the tone, is food predictive (i.e., light→tone→food; see Figure 1D; Rizley and Rescorla, 1972; Wikenheiser and Schoenbaum, 2016; Hart et al., 2020). This is because responding to the light in SPC is devaluation sensitive (Hart et al., 2020). Further, responding to the light in SPC is dependent on the status of the food-predictive tone (Rizley and Rescorla, 1972). That is, if responding to the tone is extinguished after the light and tone are presented together, the light will no longer support SPC. Thus, in contrast to much of the literature that has examined the associative structure underlying SOC, it is generally accepted that SPC produces a more complex cognitive representation of the relationships between the stimuli and outcome.

**Figure 2:**
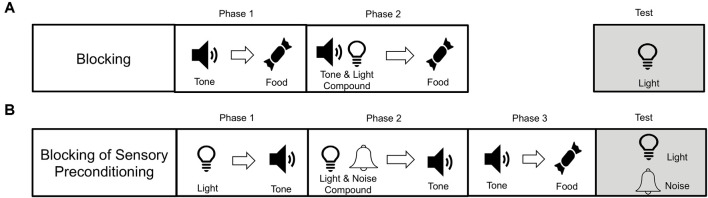
Procedural paradigms for blocking and blocking of sensory preconditioning. **(A)** Blocking involves first pairing a stimulus (e.g., a tone) with an outcome (e.g., food). Then the tone is paired in compound with another novel stimulus (e.g., light), which leads to the same food outcome (light+tone→food). Blocking is said to occur when responding to the light is reduced as a consequence of the blocking procedure. **(B)** Blocking of sensory preconditioning is when subjects first learn that two neutral stimuli are related in time (e.g., light→tone). Then the light is presented in compound with another neutral stimulus (e.g., noise), and this leads again to the tone (i.e., light+nois→tone). Like blocking with food rewards, this procedure also reduces the sensory preconditioning effect. This demonstrates that the tone can serve as a sensory-specific prediction, which can be blocked, much like a food reward that has inherent value. This supports the idea that SPC is mediated by a representation of a sensory-specific relationship between the tone and light.

Recently, Sharpe et al. (2017a) demonstrated that SPC can fall prey to the blocking effect (see Figure 2; see also Denniston et al., 1996; Blaisdell et al., 1998). In a blocking procedure (see Figure 2A), a stimulus (e.g., tone) is established as food predictive. Subsequently, the tone is presented in compound with a novel stimulus (e.g., light) and followed by food. In this example, responding to the light on a subsequent test is believed not to occur because during the compound trials the animal is already expecting food after the presentation of the tone, and so there is no violation when the tone-light compound leads to the same food. As a violation of expectations (or prediction error) is thought to be required for learning to take place (Rescorla and Wagner, 1972), learning about the light is “blocked” because it does not coincide with a prediction error. Sharpe et al. employed an SPC procedure but added an additional blocking phase (see Figure 2B). That is after the light had been paired with tone (light→tone), the light and an additional novel stimulus (e.g., noise) were paired with the tone (light+noise→tone). Again, the noise is redundant in predicting the tone. This is because the light already predicts the tone. Then, like in normal SPC, the tone is paired with food. Finally, Sharpe et al. (2017a) tested response to noise and found that it was successfully blocked, unable to promote appetitive responding. This demonstrates that neutral sensory stimuli (the light) can be used to block predictions of other neutral sensory stimuli (the noise), in a manner that transcends scalar value inherent in an outcome like food. Again, this supports the idea that training during SPC is supported by the development of sensory-specific representations between specific stimuli.

It has also been demonstrated that SPC explicitly does not involve the transfer of general value. Using a standard SPC design, where the light and tone are paired together, and then the tone is paired with food, Sharpe et al. (2017b) demonstrated that rats will not perform an instrumental response to receive presentations of the light (i.e., conditioned reinforcement). That is, the light would promote the appetitive response to go to the location where food is usually delivered, however, they would not press a lever that produced the light. This showed that the light was able to predict food, but did not become valuable in and of itself, supporting the view that SPC involved an association between the light and food, and not the light and general value, which could be achieved by virtue of the cognitive inference light→tone→food. Thus, SPC provides strong evidence that animals are capable of learning associations between various neutral stimuli which they can use to build internal models and help navigate towards rewards.

Compatible with the idea that SPC promotes the development of complex internal models of stimulus relationships, SPC recruits neural circuits that are known to play a role in these types of inferential processing, including the hippocampus and orbitofrontal cortex. For example, hippocampal neurons in CA1 increase in excitability during the pairing of the light and tone in SPC, and this excitability correlates with future response to the light after its pairing with the food-predictive tone. Further, subsequent lesions to those same stimulus-responsive neurons in CA1 disrupts responding to the light, but not the food-predictive tone (Port et al., 1987). The role of the hippocampus is also supported by studies in humans; neural activity in the hippocampus that is observed to the light during SPC is re-evoked when the tone is paired with reward, suggesting the development of the cognitive framework that supports SPC in the hippocampus (Wimmer and Shohamy, 2012). Recently, Barron et al. (2020) found that the hippocampus is not only important during light-tone pairings, but also, at the time of test, helping to support appetitive responding to the light. Specifically, optogenetic inhibition of CA1 neurons at test reduces responding to the light. Finally, areas adjacent and heavily connected to the hippocampus (e.g., retrosplenial cortex and perirhinal cortex) have been found to be necessary for the learning of stimulus-stimulus associations in SPC (Nicholson and Freeman, 2000; Robinson et al., 2011; Holmes et al., 2018; Fournier et al., 2020). Indeed, Wong et al. (2019) found that temporarily inactivating the perirhinal cortex while the tone was paired with the motivationally-significant outcome later disrupted motivated responding to the light, but not the tone. One interpretation of these data is that while the tone was paired with the outcome, the perirhinal cortex recruited a representation of the light, which was then associated with the outcome (Doll and Daw, 2016; Sharpe et al., 2017b). Thus while SPC is often thought to rely on a chain-like-association between light-tone-outcome, the perirhinal cortex might be critical in SPC procedures that promote mediated conditioning (i.e., resulting in a direct light-outcome association), and this appears to be dependent on the perirhinal cortex. In any event, these studies establish the hippocampus and several adjacent regions as critical to the development of SPC, often supporting a cognitive account of SPC but in other cases supporting the mediated account.

Similar to the role of the hippocampus in SPC, the orbitofrontal cortex is also critical to SPC. Specifically, neurons in the orbitofrontal cortex acquire responses to the light and tone during SPC in a manner that reflects the development of a sensory-specific association between the light and tone (Sadacca et al., 2018). Further, optogenetic inhibition of these neurons prevents the development of the association between the light and tone, while pharmacological inactivation of orbitofrontal cortex at test also reduces responding. This strongly implicates the orbitofrontal cortex in the stimulus-stimulus associations at play in SPC, consistent with the core function of the orbitofrontal cortex in representing and navigating through the structure of our environments (Schuck et al., 2016; Wikenheiser and Schoenbaum, 2016; Wikenheiser et al., 2017; Sharpe et al., 2019). Given the role of both the hippocampus and orbitofrontal cortex in the SPC, and their complementary roles in learning, it becomes of interest to examine how these two regions might interact to produce the complex associations that drive behavior in SPC in future research.

### Dopamine’s Role in Pavlovian and Higher-Order Conditioning

One of the modern success stories of neuroscience has been the discovery that dopamine neurons in the midbrain serve as a neural substrate for reward prediction errors that drive appetitive Pavlovian conditioning (Waelti et al., 2001; Schultz, 2016). Schultz et al. (1997) famously showed that phasic activity in midbrain dopamine neurons increases following an unexpected reward, but not when a reward is expected. For example, these neurons will exhibit a phasic response if an animal is given a reward in an unpredictable manner, but not if they have learned that a stimulus reliably predicts the delivery of the reward. This also works in the reverse. If a reward was expected but not delivered, dopamine neurons show a phasic decrease in firing from baseline. Thus, these neurons follow the mathematical patterns described in error-reduction models of associative learning (e.g., Bush and Mosteller, 1951; Rescorla and Wagner, 1972), which conceptualize learning as a process that allows our expectations to meet reality and facilitates adaptive behavior.

The content of information that can be endowed by the phasic dopamine signal has been the topic of much debate. Initially, Schultz and colleagues described the increase in dopamine firing to reflect the transfer of scalar value inherent in the reward back to a stimulus that predicts its occurrence (Schultz, 1998). This conceptualization of phasic dopamine firing is consistent with that described by the model-free temporal difference reinforcement learning (TDRL) algorithm described by Sutton and Barto (1981). Critical to this proposal is that the reward-predictive stimulus has now been endowed with value inherent in reward, and not that the stimulus is associated with a sensory-specific representation of that reward. In other terms, the reward-predictive stimulus becomes “good” but does not evoke a representation of the reward. While this value is sufficient to alter behavior to the reward-predictive stimulus (i.e., induce an appetitive response), it constrains the role that the dopamine prediction error can have in learning to value-based associations that do not comprise detailed representations between stimuli (rewarding or otherwise).

### Using Higher-Order Conditioning to Understand Dopamine’s Contribution to Learning

A number of studies have now challenged the “value hypothesis” of the dopamine prediction error (Chang et al., 2017; Sharpe et al., 2017a, 2020; Takahashi et al., 2017; Howard and Kahnt, 2018; Keiflin et al., 2019). SPC and SOC are two procedures that have helped us understand how the dopamine prediction error contributes to learning and behavior. Of course, central to the narrative that dopamine represents reward prediction error is the idea that the dopamine signal continues to back-propagate to the earliest predictor of reward. This begs the question of whether the presence of the dopamine error at the onset of a reward can support conditioning in its own right. Maes et al. (2020) confirmed this by optogenetically inhibiting dopamine neurons in the ventral tegmental area (VTA) during SOC. Rats were first trained that a tone predicted food. Then, the light was paired with the tone, and dopamine neurons in VTA were inhibited across the transition between the light and tone, to prevent a prediction error from occurring. Maes et al. (2020) found that this reduced the subsequent ability of the light to support the appetitive response, demonstrating that the dopamine prediction error can function to support the development of the light-tone pairings in SOC.

The involvement of the prediction error in SOC is consistent with it acting either as a teaching signal that facilitates the development of associations between stimuli or acting as a value signal. However, examining the role of the prediction error in SPC can dissociate between these possibilities. In fact, all error correction models of learning that rely on value to drive learning [e.g., TDRL (Sutton and Barto, 1981)], or directly-experienced outcomes (Rescorla and Wagner, 1972), have historically struggled with explaining SPC because during preconditioning there is no expectation of reward with which to generate a reward prediction error (Miller et al., 1995). Sharpe et al. (2017a) used the novel blocking of SPC described above (see Figure 2B), in combination with optogenetics, which would allow a test of whether stimulating VTA dopamine neurons could drive the sensory-specific associations present in SPC. Specifically, Sharpe et al. first paired two neutral stimuli together (e.g., light→tone; A→X), and then presented the light in compound with another novel tone stimulus, followed by the tone (AB→X). Under normal circumstances, learning about the B→X relationship is blocked because A already predicts X. However, at the transition between AB and X, they briefly stimulated VTA dopamine neurons to produce a prediction error to see whether they could unblock the B→X relationship. Consistent with this, rats receiving a prediction error during AB→X trials showed higher levels of appetitive response to the B stimulus (after × has been paired with food), relative to rats that did not receive stimulation of VTA dopamine neurons. Sharpe et al. also found that the increased appetitive response to unblocked B was sensitive to goal devaluation, demonstrating that the presence of the dopamine prediction error endowed rats with a sensory-specific association between B→X that allowed B to become predictive of the specific food reward predicted by X.

The nature of SPC also facilitates an examination of whether dopamine can “add” value to an antecedent stimulus, as well as endowing a cognitive representation of stimulus transitions (e.g., light→tone). Recall, SPC does not endow the neutral, “preconditioned cue” (e.g., the light, A, or B) with a general value that supports conditioned reinforcement. Sharpe et al. (2020) used this premise to test whether optogenetic stimulation of dopamine neurons would allow the preconditioned cue to gain value that would promote conditioned reinforcement. That is, rats first experienced A and X paired together (A→X), and then the compound AB was paired with X, during which a prediction error was produced using optogenetics to unblock the B→X association. Here, rats showed higher levels of response into the food port when B was presented, showing dopamine unblocked the B→X association as previously demonstrated, but they would not press a lever to receive B. This demonstrates that stimulation of dopamine neurons facilitated the sensory-specific associations present in SPC, without adding value to these associations. These data are consistent with a role for the dopamine prediction error in acting as a teaching signal to drive associations between stimuli, and not as a signal that makes antecedent stimuli valuable.

## Discussion

### Extended Role of Higher-Order Conditioning (and Potentially Dopamine) in Cognition

Midbrain dopamine neurons have now been causally implicated in both SOC and SPC (Sharpe et al., 2017a; Maes et al., 2020). While their involvement in SOC is not unexpected, that they’re critical to the formation of the stimulus-stimulus association in SPC is surprising. This is because it positions dopamine to facilitate Pavlovian conditioning in a more flexible manner than previously conceptualized. Further, that these higher-order phenomena are associatively and neurologically distinct, and yet both fundamentally driven by dopamine, demonstrates that the role of dopamine prediction errors in learning need not be constrained by specific associative or neurological structures. Put another way, while dopamine was once thought to act as a value signal, which restricts the role it can play in associative learning, its involvement in higher-order conditioning processes suggests a much broader role for dopamine as a critical driver of Hebbian plasticity in many regions of the brain.

What are the implications of dopamine being involved in learning in such a broad way? To understand this, we need to think about the more general role of higher-order stimulus relations play in complex behavior and cognitive processes. For instance, Blaisdell and colleagues have explored the role of SPC in forming cognitive maps for spatial search (Blaisdell and Cook, 2005; Sawa et al., 2005; Bouchekioua et al., 2021). In one experiment, pigeons were taught a consistent relationship between visual landmarks on a 4 × 4 grid of gravel-filled cups (e.g., Landmark 2 is always two cups to the left of Landmark 1). Then, pigeons were separately taught a relationship between Landmark 1 and the hidden location of food (e.g., food is always one cup below Landmark 1). At the test, pigeons were presented with Landmark 2, and they were able to locate the food despite never having experienced the relationship between Landmark 2 and the food cup (Blaisdell and Cook, 2005). Similar results were obtained with pigeons using a modified version of this task using an operant touchscreen (Sawa et al., 2005), a computer version in humans (Molet et al., 2010), and the Morris water maze with rats (Chamizo et al., 2006). At present, there has been little investigation of the neural basis of the integration of these separately learned spatial maps, but it is exciting to think that dopamine may be critical for such sophisticated cognitive processes. Indeed, mice lacking D_1_ dopamine receptors showed deficits in several spatial learning tasks without showing deficits in visual or motor performance (El-Ghundi et al., 1999).

There is also evidence for the integration of temporal maps in higher-order conditioning procedures. The temporal coding hypothesis describes the role time plays in associative learning experiments (Miller and Barnet, 1993; Savastano and Miller, 1998; Arcediano et al., 2003). Analogous to the role of higher-order conditioning in the integration of spatial maps, temporal maps acquired during Pavlovian conditioning can be integrated as a result of higher-order conditioning procedures. In one example, Leising et al. (2007) presented rats with a long (60 s) light paired with a short (10 s) tone^1^. However, one group of rats had the tone onset soon after the onset of the light (“group early”), thus it terminated well before the light terminated. The tone for the other group onset toward the end of the light presentation (“group late”). The tone was then paired with food, and appetitive response was examined to the light. Appetitive response was higher at the beginning of the light in the group early, relative to the group late. Similar results have been reported using fear conditioning procedures in rats (Savastano and Miller, 1998) and appetitive procedures in humans (e.g., Arcediano et al., 2003). This research demonstrates that rats had not only encoded the relationships between the light and tone but that they encoded these relationships into a temporal map. Again, it would be interesting to think about how dopamine might contribute to the inferred temporal relationships that can be formed during the SPC procedure.

Higher-order associative processes even appear to be involved in learning causal models of events. In a study using appetitive SPC, Blaisdell et al. (2006) showed rats can infer different causal models by integrating associations between the light, tone, and food (see also Leising et al., 2008). For instance, if rats are taught to encode a causal chain model whereby light→tone→food^1^, they will expect the delivery of food: (1) if they press a lever to receive presentations of a light, or (2) if the light is presented alone without a lever press. However, if they are taught that the tone produces both the light and the food (i.e., rats learn that tone→light and also that tone→food), they will show appetitive response to the light when presented without a lever press, but not when the light was caused by a lever press. This is because they reason that, in the latter case, the light was caused by their own action and not by the tone, as it was in the former case. Thus, they did not expect the light to produce a food reward. This sophisticated reasoning process exhibited by these rats is akin to that observed in adults (e.g., Waldmann and Hagmayer, 2005) and children (e.g., Gopnik et al., 2004). These results and others (e.g., Dwyer et al., 1998) illustrate the far-reaching involvement of higher-order conditioning processes in many aspects of cognition. However, there is a dearth of research on the role of dopamine—or other neural substrates—in these domains.

What is next for those interested in understanding how dopamine and higher-order processes give rise to more complex cognition? One direction is that these sophisticated learning procedures could be coupled with recently developed technologies to record from and manipulate dopamine and related circuits. Because these techniques (e.g., optogenetics, calcium imaging) allow access to specific neuronal cell types and their projections and have a high degree of temporal specificity, they can be used to understand how distinct neuronal populations contribute to higher-order conditioning, as well as identify circuits between various regions that are involved in these processes, over very short timescales (Deisseroth, 2011; Patriarchi et al., 2018; Sych et al., 2019). This increase in specificity is critical to understanding the anatomical and associative basis of SOC and SPC. Similarly, while the circuits that support learning of neutral stimuli in SPC are ongoing, there is also recent evidence that some regions (e.g., Lateral Hypothalamus) might actively oppose the development of neutral associations that underlie SPC (Hoang and Sharpe, 2021; Sharpe et al., 2021). This brings to bear the possibility that there is more than one system at play in the forming of these associations. More generally, future research utilizing these tools in combination with higher-order tasks would help to elucidate how we make sense of the world around us, and how this may go awry in psychological disorders.

## Author Contributions

All authors contributed to the synthesis of research and writing of the article. All authors contributed to the article and approved the submitted version.

## Conflict of Interest

The authors declare that the research was conducted in the absence of any commercial or financial relationships that could be construed as a potential conflict of interest.

## Publisher’s Note

All claims expressed in this article are solely those of the authors and do not necessarily represent those of their affiliated organizations, or those of the publisher, the editors and the reviewers. Any product that may be evaluated in this article, or claim that may be made by its manufacturer, is not guaranteed or endorsed by the publisher.

## Footnotes

^1^Note in the studies conducted by Sawa et al. (2005) and Blaisdell et al. (2006), tone and light were used in the opposite manner. That is, in Sawa et al., the tone was the longer 60 s cue and in Blaisdell et al., the tone was the common cause of light and food. We have revised our descriptions of these studies so that tone remains the cue directly paired with food as is consistent with all other examples in this article.
